# Mucosal and systemic immune responses after a single intranasal dose of nanoparticle and spore-based subunit vaccines in mice with pre-existing lung mycobacterial immunity

**DOI:** 10.3389/fimmu.2023.1306449

**Published:** 2023-12-07

**Authors:** Emil Joseph Vergara, Andy Cano Tran, Mi-Young Kim, Tufária Mussá, Matthew J. Paul, Thomas Harrison, Rajko Reljic

**Affiliations:** ^1^ Institute for Infection and Immunity, St. George’s University of London, London, United Kingdom; ^2^ Department of Molecular Biology, Jeonbuk National University, Jeonju, Republic of Korea; ^3^ Department of Microbiology, Faculty of Medicine, Eduardo Mondlane University, Maputo, Mozambique

**Keywords:** tuberculosis, vaccine, post-exposure vaccine, mucosal vaccination, T cells, antibodies, dendritic cells, adjuvants

## Abstract

Tuberculosis (TB) is a major global health threat that claims more than one million lives annually. With a quarter of the global population harbouring latent TB, post-exposure vaccination aimed at high-risk populations that could develop active TB disease would be of great public health benefit. Mucosal vaccination is an attractive approach for a predominantly lung disease like TB because it elicits both local and systemic immunity. However, the immunological consequence of mucosal immunisation in the presence of existing lung immunity remains largely unexplored. Using a mycobacterial pre-exposure mouse model, we assessed whether pre-existing mucosal and systemic immune responses can be boosted and/or qualitatively altered by intranasal administration of spore- and nanoparticle-based subunit vaccines. Analysis of lung T cell responses revealed an increasing trend in the frequency of important CD4 and CD8 T cell subsets, and T effector memory cells with a Th1 cytokine (IFNγ and TNFα) signature among immunised mice. Additionally, significantly greater antigen specific Th1, Th17 and IL-10 responses, and antigen-induced T cell proliferation were seen from the spleens of immunised mice. Measurement of antigen-specific IgG and IgA from blood and bronchoalveolar lavage fluid also revealed enhanced systemic and local humoral immune responses among immunised animals. Lastly, peripheral blood mononuclear cells (PBMCs) obtained from the TB-endemic country of Mozambique show that individuals with LTBI showed significantly greater CD4 T cell reactivity to the vaccine candidate as compared to healthy controls. These results support further testing of Spore-FP1 and Nano-FP1 as post-exposure TB vaccines.

## Introduction

Tuberculosis (TB) is a major global health threat that claims more than one million lives annually (1). Post-exposure vaccination is an attractive strategy to help address the global burden of TB. This approach is aimed at already individuals infected with *Mycobacterium tuberculosis* (Mtb) to either prevent activation of latent TB infection (LTBI) to active TB disease (aTB), work alongside antibiotic therapy to increase cure rates and/or reduce treatment duration, or lower the incidence of disease relapse for treated aTB patients (2). Current estimates show that a quarter of the global population harbour LTBI. Among these, roughly 5-10% will develop active TB disease with individuals who have co-infection with HIV at a significantly greater risk. Thus, vaccines targeting latently infected individuals to drastically reduce disease activation is one of the key goals in TB vaccine development.

There is currently no licensed vaccine for TB apart from Bacille Calmette-Guerin (BCG), which has been in use for more than a century to prevent disseminated and miliary TB disease in infants and children (3). Subunit vaccines are an attractive modality for TB because of the relatively low cost of production and favourable safety profile, including among individuals with HIV. Additionally, it has been proposed that subunit vaccines, unlike whole cell-based vaccines, can improve immune responses in BCG immunised individuals in populations highly sensitised to environmental mycobacteria (4). Great strides have been achieved in the past decades in advancing several candidates to the clinical phases of vaccine development. M72/AS01, a post-exposure subunit vaccine containing two Mtb antigens (Mtb32A and Mtb39A) and Glaxo Smith Kline’s proprietary adjuvant AS01, demonstrated 49.7% efficacy in preventing reactivation in LTBI participants after a 36 month follow up (5). Efforts are underway to demonstrate vaccine efficacy in a phase 3 clinical study across different centres (6).

Intranasal vaccination stimulates both mucosal and systemic immune responses, thus making it an attractive strategy for respiratory pathogens (7). For respiratory diseases like TB, it is thought that eliciting local lung immune responses, in the form of tissue resident T cells and mucosal antibodies, may prove beneficial for restricting mycobacterial growth within the lungs (8). Additionally, it was shown that unlike the parenteral route of immunisation, pulmonary delivery of a vaccine can circumvent tolerogenic responses induced by environmental mycobacteria (9). This led to many efforts at developing mucosally administered vaccines to target Mtb. So far, only AdHu5Ag85A, an adenovirus vectored vaccine, has successfully moved forward and completed a phase I clinical trials where it demonstrated that aerosol administration was successful at eliciting antigen-specific T cell responses in the circulation and, more importantly, the airways (10).

Mtb infected individuals exhibit a plethora of antigen specific immunity in the lungs as evidenced by previous studies showing PPD-specific airway T cell responses in active TB patients and healthy household contacts (11, 12). One unexplored facet of TB vaccinology is whether vaccine delivered through the respiratory mucosa can generate favourable immune responses in an immune environment where immunity elicited by Mtb may dominate the local milieu. In a mouse model of latent Mtb infection, lung resident T cell subsets (CD44^hi^CD62L^low^CD69^+^ and CD44^hi^CD62^low^ CD4 T cells) and Ag85B-specific T cells remain significantly increased among infected mice for as long as 7 months after infection (13). In humans, one study has shown that as much as 10% of CD4 T cells in the broncho-alveolar lavage fluid (BALF) of people from Malawi were reactive to purified protein derivative (PPD) (14) whereas another showed significantly greater IFNγ-positive BALF lymphocytes among subjects that had no history of BCG vaccination but were PPD skin test positive (15).

Previously, it has been shown that strong mycobacterial T cell responses are elicited by either aerosol infection with pathogenic Mtb or intratracheal administration of BCG in mice (16). Here, we used BCG infection to model local and systemic immune responses in mice. BCG was used as a proxy to Mtb to replicate mycobacterial immunity in LTBI without the immunopathology seen in active TB disease. Using a single intranasal dose of two protein subunit vaccines, Spore-FP1 (17) and Nano-FP1 (18), we demonstrate that favourable T cell responses in the lungs and spleens can be further increased. Additionally, we also observed higher titres of antigen-specific antibodies in both systemic and mucosal compartments of immunised mice. Lastly, PBMCs obtained from individuals with LTBI showed significantly higher CD4 T cell reactivity to the vaccine candidate as compared to healthy controls.

## Materials and methods

### Ethics statement

Mouse experiments were conducted in compliance with the Animal (Scientific Procedures) Act, 1986, and with approval from London School of Hygiene and Tropical Medicine Ethics committee under Home Office animal project license 70/7490. Human blood collected from healthy donors from the National Health Service Blood and Transplant Unit (NHSBT) at St. George’s Hospital London under ethical approval SGREC16.0009. Studies on PBMCs from the latent TB infected and healthy cohort in Mozambique were approved by the Ministry of Health Committee of Bioethics and Health ref 298/CNBS/15 as part of the EU Horizon, 2020-funded project EMI-TB (643558). Blood was collected from LTBI participants and healthy controls in the Maputo Region (Mozambique), using standard operating procedures. The samples in this study were collected after receiving written informed consent from each participant over the age of 18 or through a guardian or parent for participants under 18 years old.

### Mice and immunisations

Female BALB/c mice 6 – to 8-weeks of age were purchased from Charles River. After an acclimatisation period of 7 days, mice were randomly housed in cages with each cage corresponding to each treatment group. Prior to intranasal infection or immunisation, mice were sedated with a cocktail of Xylazine (10mg/kg) and Ketamine (80mg/kg) delivered intraperitoneally. Once sedated, animals were given 100µL (50µL/nostril) of either sterile DPBS or BCG (5x10^5^ CFUs/animal; resuspended in DPBS) using a 200µL pipettor. After 4 weeks, mice were given either PBS, heat-inactivated *Bacillus subtilis* spores, lipidic nanoparticle (YC-NaMA, herein called Nano), Spore-FP1 or Nano-FP1 intranasally. Mice were sacrificed after three weeks. Blood, bronchoalveolar lavage fluid, spleens and lungs were collected from each mouse. All mice used in the experiments were kept under specific pathogen free (SPF) conditions at the Biological Resource Facility (BRF) of London School of Hygiene and Tropical Medicine. Mice were provided *ad libitum* access to standard rodent diet and water.

### Vaccine formulation

Formulation of Spore-FP1 and Nano-FP1 was done as previously described (18, 19). First, FP1 was incubated with heat inactivated *Bacillus subtilis* spores (Sporegen, UK) or YC-NaMA (Particle Sciences, USA) for 1 hour at room temperature. Poly(I:C) (InvivoGen, tlrl-pic) was added to vaccine formulations right before immunisation. Vaccines were formulated so that each animal received 0.1% (w/v) YC-NaMA, or 1x10^8^ *B. subtilis* spores with 10µg FP1 and 20µg poly(I:C) all in sterile Dulbeco’s phosphate buffered saline (Sigma, D8547).

### Culture and use of laboratory strains of mycobacteria

*Mycobacterium bovis* BCG Pasteur used in the study are cultures maintained at the Institute for Infection and Immunity, St. George’s, University of London. Bacteria were propagated in Difco™ Middlebrook 7H9 Broth (BD,271391) supplemented with 10% v/v Middlebrook 7H10 oleic acid, albumin, dextrose, and catalase (OADC) (USBiological Life Sciences, M3895-01). All mycobacterial cultures were propagated for up to 3 weeks. Frozen stocks were made with sterile deionised distilled water with 10% glycerol kept at -80°C.

### Mycobacterial vaccine antigens

Ag85B, Acr and FP1 are all *E. coli* expressed proteins obtained from Lionex (Braunscweig, Germany). To verify protein quality, stock solutions were loaded onto SDS-PAGE followed by Coomassie blue staining. Protein concentrations were verified through measurement of A280/A260 using a spectrophotometer (NanoDrop, 2000, Thermo Scientific).

### Processing of bronchoalveolar lavage fluid

BALF were initially centrifuged at 300 rcf for 5 minutes in a cold centrifuge to obtain the cell fraction. Afterwards, the fluid was further centrifuged at, 9000 rcf for 10 min in 4°C. The supernatant was collected and stored at -20°C before processing. For the measurement of antigen-specific IgA and IgG, BALF was concentrated using Amicon^®^ 50kDA centricon tubes (Millipore, UFC5050). For this, 250µL of neat BALF was added to centricon tubes followed by cold centrifugation at, 9000 rcf for 30 min. Concentrated fractions were reconstituted to a final concentration of 50µL (5x concentrated BALF).

### Processing whole blood to obtain serum

Whole blood collected from mice was left to clot at room temperature. Tubes were then centrifuged at, 9000 rcf for 10 min to separate the serum from cell components. After collecting the cell-free fraction, the sample was again centrifuged at, 9000 rcf for 10 min. Serum was separated from the remaining cells and debris by pipetting and stored at 20°C before processing.

### Measuring antigen-specific antibodies in BALF and serum

Previously described in-house direct ELISAs were used to detect antigen-specific IgG and IgA from serum and BALF (19). Briefly, 100µL of antigen in PBS (5µg/mL Ag85B or Acr) were added to 96-well Nunc-Immuno plates (Thermo Scientific, 442404) and incubated in 4°C overnight. After blocking with 5% skim milk for 2 h in room temperature, serial dilutions of serum or BALF were added followed by overnight incubation at 4°C. Goat anti-mouse IgA HRP (Invitrogen, 62-6720) or anti-mouse IgG peroxidase (Sigma, A2554) (both 1:10,000 in 5% skim milk) was added to each well and incubated at room temperature for 1 h. Signals were developed through the addition of TMB substrate (Invitrogen, 00-4201-56) followed by a stop solution (0.16M H_2_S0_4_, in-house). Absorbance reading for each plate was obtained at 450nm with a correction of 540nm using a plate reader (Tecan, UK).

### Primary cell culture

Single cell suspensions from spleens and lungs were maintained complete Roswell Park Memorial Institute (RPMI) – 1640 Medium (Sigma, R0833) (hereby referred to as R10 media). R10 media is composed of 10% Foetal Bovine Serum (FBS), (Sigma-Aldrich, F9665), 5mM L-Glutamine (Sigma, G7513), 100 U/mL Penicillin & 100µg/mL Streptomycin (Sigma, P4333), 50µM β-mercaptoethanol (Sigma, M3148), and 10mM (4-(2-hydroxyethyl)-1-piperazineethanosulfonic acid (HEPES), buffer (Gibco, 15630-056). Cell cultures were kept in a humidified CO2 incubator (5% CO_2_ and 37°C).

### Generation of single cell suspensions from mouse spleen and lung

Spleen single cell suspensions were obtained through mechanical disruption of the spleen with the flat end of a syringe barrel through a prewashed 70µm cell strainer (Falcon, 352350) in a 50mL Falcon tube. After rinsing the cell strainer with 10mL R10 media, tubes were centrifuged at 300 rcf for 5 min. After discarding the supernatant, red blood cells (RBCs) were lysed through the addition of 2mL ACK lysis buffer (Gibco, A10492-01), with incubation at room temperature for 5 min. Lysis was stopped through the addition of 18mL R10 media and cell were again centrifuged at 300 rcf for 5 min. Pelleted cells were resuspended in R10 media counted through Trypan Blue exclusion method. Single cell suspensions of lungs were obtained by first mechanically macerating the lung tissue to smaller pieces (around 1mm) on a plastic Petri dish using a sterile scalpel. Macerated tissue was rinsed with 1mL DPBS (with Magnesium and Calcium) and transferred to a 50mL conical tube. Tissues were enzymatically dissociated through the addition of 1mL DNAse/collagenase enzyme cocktail for a final concentration of 150µg/mL DNAse (Roche, 10104159001) and 1mg/mL collagenase (Gibco, 1710101). Tubes were left in a 37°C shaking incubator for 40 min. Afterwards, 8mL of R10 medium was added to arrest enzyme activity followed by pouring the contents of the tube to a 70µm cell strainer in a 50mL Falcon tube. The lung tissue was mashed using the flat end of the syringe barrel followed by with 10mL R10 media. Cells were centrifuged at 300 rcf for 5 min, followed by a red blood cell lysis step. Finally, cells were resuspended in R10 medium and counted using Trypan Blue exclusion method.

### Short-term and long-term antigen recall assays with mouse lung and spleen single cell suspensions

Antigen recall experiments were done as previously described (19). Short-term antigen recall (24 hours of simulation) experiments were performed to investigate the frequency of antigen-specific T cells while long-term antigen recall (72 hours of stimulation) experiments were done to measure Th1 cytokine profile, T cell proliferation and cytokine secretion after sustained antigen stimulation. Briefly, 1x10^6^ cells were plated on 96-well U bottom (short-term) (Corning, 353077) or flat-bottom (long-term) (Corning, 353072) plates and were left unstimulated (RPMI medium alone) or stimulated with 5µg/mL of individual antigens (Ag85B or Acr) or 10 µg/mL fusion protein (Ag85B-Acr-HBHA). Plates for short-term antigen recall were cultured at 100µL/well while plates for long-term antigen recall were cultured at 250 µL/well. Cell suspensions were then incubated for either 24 hours or 72 hours. For both recall assays, cells were treated with 5µg/mL Brefeldin A (Biolegend, 420601) 6 hours prior to harvest. Cells were processed for surface and intracellular staining and FACS acquisition. Culture supernatants were obtained from long-term antigen recall experiments and kept frozen at -80°C until processing.

### Antigen recall assay using human PBMCs

Cryopreserved PBMCs were revived by quickly thawing vials in a warm bath set to 37°C, then transferring the contents to a 50mL Falcon tube with 20mL R10 media with 150µg/mL DNase I. After centrifugation (1500rpm for 5 min) and discarding the supernatant, cells were washed twice with complete RPMI-1640. Cell count and viability were assessed using Trypan Blue exclusion method. Single cell suspensions PBMCs were seeded onto 96 flat-bottom well plates at a density of 1 million cells per well. Cells were stimulated FP1 (5ug/mL). Additionally, PBS and PMA (10ng/mL; Sigma, P1585)/ionomycin (250ng/mL; Sigma, I9657) were used in negative and positive control cultures, respectively. Plates were left in the CO2 incubator for 18-24 h. After incubation, plates were centrifuged at 500 rcf for 5 min and supernatants were discarded, and cells surface and intracellularly stained for flow cytometry analysis.

### Monocyte-derived dendritic cell differentiation and activation

CD14+ monocytes were enriched from cryopreserved PBMCs obtained from healthy donors using MojoSort™ Human Pan Monocyte isolation kit (BioLegend^®^, 480060) according to the manufacturer’s recommendations. Enrichment from PBMCs yielded >90% purity and >95% viability of CD14+ monocytes (Data not shown). To differentiate CD14+ monocytes to immature moDCs, we used a commercially available dendritic cell generation medium (PromoCell^®^, C-28050). For this, enriched cells were plated on 24-well tissue culture plates (Corning, 343047) at a density of 1x10 (6) cells per well and left to differentiate through the addition of cytokine cocktails in the media for 6 days. At day 6, immature moDCs were harvested and plated (50,000 moDCs/well) on 96-well U bottom plates and were either left untreated or treated with lipopolysaccharide (LPS) (100ng/mL) (Sigma, L4516), Poly(I:C) (20µg/mL) or delivery systems (Spore or YC-NaMA) with or without Poly(I:C). After 48 h, culture supernatants were collected and frozen at -80°C, while cells were processed for analysis of activation-induced cell surface markers using flow cytometry.

### Membrane and intracellular staining for Flow cytometry analysis

Single cell suspensions cells were transferred to 96-well U-bottom plates and centrifuged at 500 rcf for 5 minutes. Cells were washed with sterile PBS thrice, then stained using a cocktail of antibodies (1:200) (**Table 1**), fixable viability marker (1:500) (Invitrogen, 65-0865-14), and human/mouse Fc block (Human TruStain FcX™/TruStain fcX™ [anti-mouse CD16/32]) (1:250) (Biolegend^®^, 422302) for 30 minutes at 4°C. Afterwards, cells were washed twice then resuspended in sterile 1X PBS prior to FACS acquisition and analysis using CytoFlex (BD). Where applicable, intracellular staining was done after membrane staining of cells. Initially, IC fixation buffer (Invitrogen, 00-822-49) was added to cells. Plates were incubated in the fridge (4°C) for 20 minutes, then washed twice with 1X permeabilization buffer (Invitrogen, 00-8333-56). After washing, pelleted cells were stained with antibody cocktails diluted in 1X permeabilisation buffer for 30 minutes in the fridge. Cells were washed twice with 1X permeabilization buffer, then resuspended in sterile 1X PBS prior to FACS acquisition and analysis using CytoFlex (Beckman Coulter). Before running samples, compensation for spectral overlap between different channels of CytoFlex was performed with the use of compensation beads (VersaComp Antibody Capture Bead Kit) (Beckman Coulter, B22804). Compensation adjustments were automatically done through CytExpert Software (Beckman Coulter) post-acquisition of compensation beads + fluorochrome conjugated antibodies. Compensation calculations were applied prior to acquiring samples. Optimization of gating strategy was done using fluorescent minus one (FMOs) on cells of interest wherein all fluorochrome-labelled antibodies were added to the sample except for the marker of interest. Populations are then gated in dot plots or histograms based on negative populations. Flow cytometry results were analysed using FlowJoTM v.10.8 Software (BD Life Sciences). Dimensionality reduction with t-stochastic neighbour embedding (tSNE) algorithm was performed using the built-in plugin in FlowJoTM v.10. We followed the same workflow as the one described online (https://www.flowjo.com/blog/post/dimensionality-reduction-t-distributed-stochastic-neighbor-embedding-tsne-algorithm) for generating tSNE plots.

**Table 1:** Fluorochrome-conjugated antibodies used for flow cytometry.

	Marker	Clone	Fluorochrome	Reference Number	Source
Mouse	CD3	17A2	APC	100236	BioLegend®
	CD4	GKI.5	PerCP/Cyanine5.5	100434	BioLegend®
	CD8	53-6.7	Brilliant Violet 510™	100751	BioLegend®
	CD44	IM7	FITC	103006	BioLegend®
	CD62L	MEL-14	PE	104408	BioLegend®
	CD69	H1.2F3	PE/Cyanine7	104512	BioLegend®
	CD103	2.00E+07	Brilliant Violet 421™	121422	BioLegend®
	IFNγ	XMG1.2	PE/Dazzle™ 594	505846	BioLegend®
	TNFα	MP6-XT22	PE/Cyanine7	506324	BioLegend®
	Ki-67	16A8	Brilliant Violet 605™	652413	BioLegend®
Human	Marker	Clone	Fluorochrome	Reference Number	Source
	CD3	OKT3	Brilliant Violet 421™	317344	BioLegend®
	CD4	A161A1	PERCP/Cyanine5.5	357414	BioLegend®
	CD8a	RPA-T8	Brilliant Violet 510™	301048	BioLegend®
	IFNγ	B27	Alexa Fluor® 700	557995	BD Pharmingen
	TNFα	MAb11	PE/Cyanine7	502930	BioLegend®
	HLA-DR	LN3	PE/Cyanine7	25-9956-42	Invitrogen
	HLA-ABC	W6/32	PerCP-eFluor™710	46-9983-42	Invitrogen
	CD80	2D10	Brilliant Violet 510™	305234	BioLegend®
	CD86	IT2.2	PE/Cyanine7	305421	BioLegend®
	CD40	5C3	PE/Cyanine7	334322	BioLegend®
	CCR7	G043H7	Brilliant Violet 421™	353208	BioLegend®

### ELISA using cell culture supernatants

Commercially available kits (all from Invitrogen) were used to quantify IFNγ (88–7314–88), TNFα (88–7324–88), IL-10 (88–7105–88), and IL-17A (88–7371–88) from culture supernatants as per the manufacturer’s recommendations. Briefly, culture supernatants were diluted 1:5 (TNFα, IL-10, and IL-17A) or 1:20 (IFNγ). Dilutions used were based on an optimisation experiment which aimed to ensure that cytokine concentrations in samples fall within the standard curve of each kit. Plates were initially coated with 100µL capture antibody in PBS overnight at 4°C. After three wash steps with PBS + 0.05% Tween-20 (PBS-T), wells were blocked with 200µL blocking buffer for 1 hour at room temperature. Wells were then washed three times, then 100µL of diluted samples or serial dilutions of the standard were added to wells. After two hours of incubation at room temperature, wells were washed five times with PBS-T, followed by the addition of 100µL of detection antibody. Plates were incubated at room temperature for an hour and washed five times with PBS-T. Wells were then incubated for 1 hour at room temperature with 100µL of streptavidin-HRP. After repeat washes with PBS-T, TMB substrate was added to each well and plates were left to develop for 15 minutes, followed by the addition of a stop solution (In-house). Absorbance reading for each plate was obtained at 450nm with a correction of 540nm using a plate reader (Tecan, UK). Absorbance reading from blank wells were subtracted from all wells with samples or standard.

### LEGENDplex™ assay to measure inflammatory cytokines

To quantify secreted soluble mediators by dendritic cells, we used LEGENDplex™ (BioLegend®, 740808) which is a flow cytometry based 13-plex multi-analyte kit able to detect IL-1β, IFN-α2, IFNγ, TNFα, IL-6, IL-18, IL-10, IL-17A, IL-12p70, IL-23, IL-33, CCL2 and CXCL8. Equal volumes of assay buffer, culture supernatants and capture beads were loaded onto wells of a 96-well V bottom plate and left to incubate at room temperature on a plate shaker set to 300 rcf. Afterwards, plates were centrifuged at 250 rcf and washed with assay buffer. Wells were incubated for 1 hour at room temperature with detection antibodies, followed by the addition of biotinylated detection antibody. After an additional 30 minutes of incubation, plates were centrifuged at 250 rcf and washed with assay buffer. Wells were resuspended in wash buffer prior to FACS acquisition using CytoFlex (BD). The machine was set up according to the kit manufacturer’s recommendations using setup beads. Values for each cytokine was obtained using the LEGENDplex™ data analysis software (biolegend.com/en-us/Legendplex).

### Statistical analysis

All statistical analyses were performed in GraphPad Prism Version 10.0.2 (La Jolla, CA). Where applicable, unpaired t-test, one-way ANOVA by Dunnett’s or Tukey’s test for multiple comparisons, or two-way ANOVA followed by Tukey’s range test as *post-hoc* analysis was done. Statistical tests performed are described in corresponding figure legends. *P < 0.05, **P < 0.01, ***P < 0.001, and ****P<0.0001 were considered significant.

## Results

### Characterisation of lung T cell responses after intranasal administration of Spore-FP1 and Nano-FP1 in mice with pre-existing mycobacterial immunity

**Figure 1:**
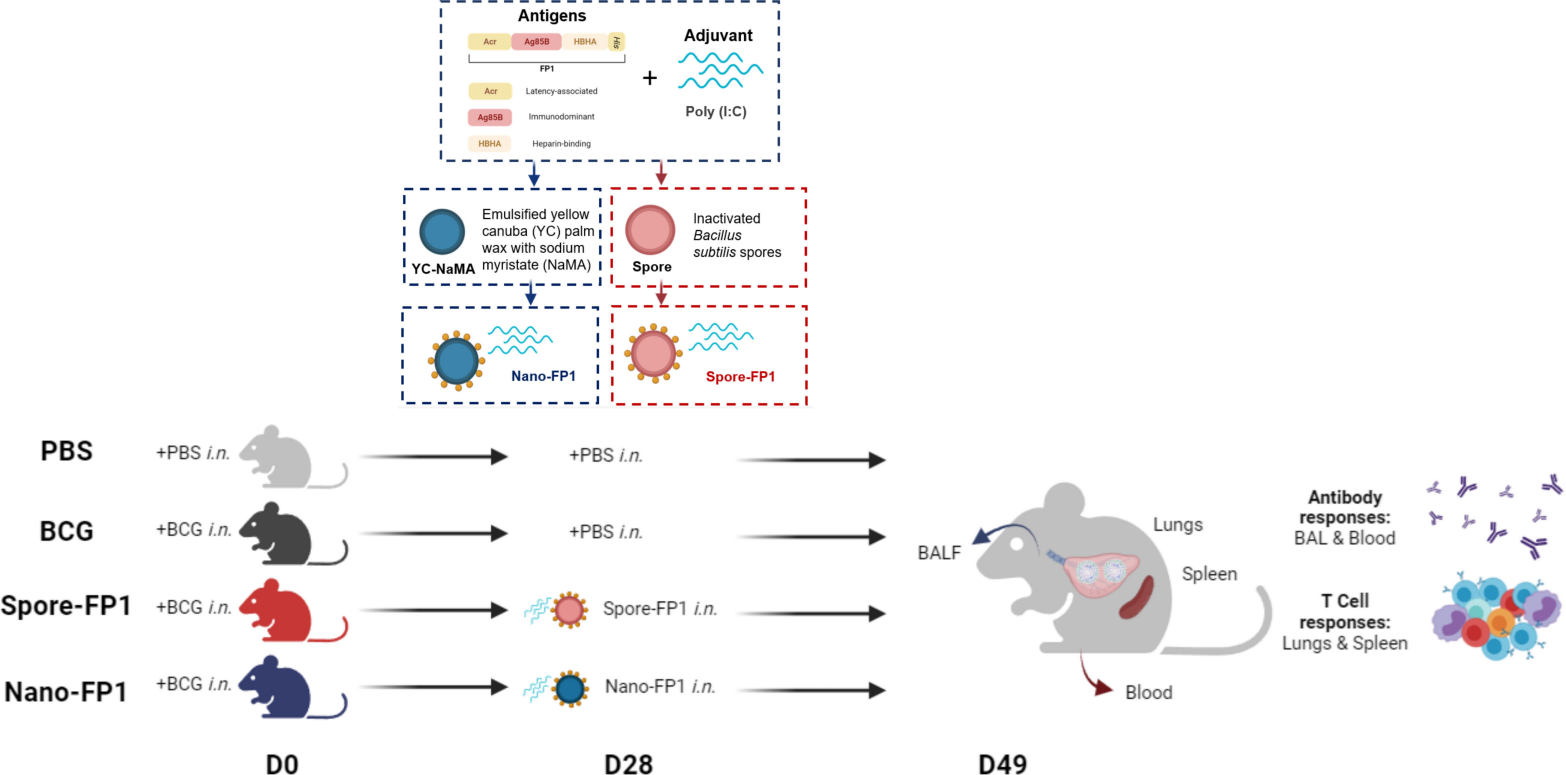
Overall experimental design to assess the immunogenicity of Spore-FP1 and Nano-FP1 as post-exposure mucosal vaccines in mice. Each mouse was first intranasally infected with 5x10^5^ CFUs of BCG. After four weeks, animals received either PBS or Vaccine candidates (Spore-FP1 or Nano-FP1). After three weeks, lungs and spleens were collected from mice to probe cellular immune responses in the mucosal and systemic compartments, respectively. BALF and serum were also obtained to quantify mucosal and systemic antigen-specific antibodies, respectively.

**Figure 2:**
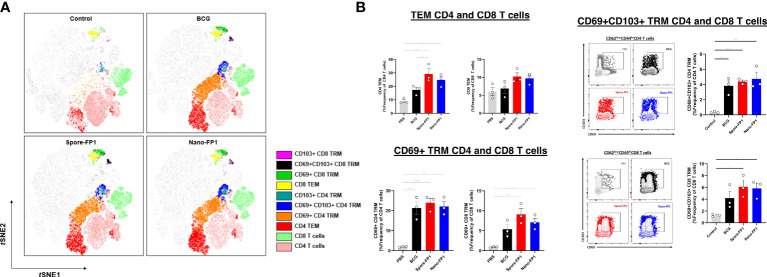
Increased CD4 and CD8 T cell subsets in the lungs after intranasal immunisation with vaccine candidates. Lungs collected from mice (n=3/group) three weeks after immunisation were processed to obtain single cell suspensions which were stained for CD4 and CD8 T cell subsets. Tissue resident memory (TRM) T cells were defined as CD44+CD62L-CD69+CD103+, CD44+CD62L-CD69+CD103- or CD44+CD62L-CD69+CD103+ whereas T effector memory as CD44+CD62L-CD69-CD103-. **(A)** tSNE plots of T cell subsets across all groups coloured based on identified clusters. **(B)** Representative dot plots and bar graphs showing the frequency of T cell subsets in the PBS, BCG, Spore-FP1 and Nano-FP1 groups. All bars are expressed as means +/- SEM. One-way ANOVA followed by Tukey’s multiple comparisons test was used to compare the groups. P<0.05 = *; P<0.01 = **; P<0.001 = ***.

Spore-FP1 and Nano-FP1 were both tested as mucosal prophylactic TB vaccines to boost parenteral BCG in mouse models (18, 19). To ascertain whether Spore-FP1 and Nano-FP1 can be used as post-exposure mucosal vaccines, we designed an experiment wherein mice inoculated with BCG intranasally were, after a four-week rest, immunised with Spore-FP1 and Nano-FP1 through the same route. We asked whether the vaccine candidates can increase the frequency of potentially protective T cell subsets in the lungs and spleen, as well as antigen-specific titres in the blood and BALF (**Figure 1**). In the lungs, we specifically probed for the presence of effector memory (TEM), tissue resident memory (TRM) and Th1 cytokine expressing CD4 and CD8 T cells. We defined T cell subsets of interest based on cell surface markers (**Supplementary Figure 1A**) through flow cytometry. First, we observed a significantly higher number of cells in the lungs of mice that received BCG intranasally compared to naïve mice (P<0.01), with the Spore-FP1 and Nano-FP1, as well as the vaccine carriers themselves not significantly affected the cell balance, apart from the spores alone inducing a further small increase (**Supplementary Figure 2A**). Similar trends were observed for both CD4 and CD8 subsets (**Supplementary Figure 2B**). Dimensionality reduction shows a strong enrichment of certain CD4+ and CD8+ T cell subsets such as CD4 and CD8 TEM and TRM cell subsets in the lungs of mice that were infected BCG (**Figure 2A**). Further analysis revealed that mice which received Spore-FP1 and Nano-FP1 showed significantly greater frequencies of CD4 TEM, CD69+ CD8 TRM and CD69+CD103+ CD8 TRM subsets than the PBS group, which was not the case for mice which received BCG alone (**Figure 2B**). To see whether the increase in lung T cell subsets were not due to the vaccine delivery systems (Spore/Nano) with Poly(I:C) (herein referred to as Spore or Nano), we compared lung T cell responses of mice that received either Nano/Spore alone or Spore-FP1/Nano-FP1. Overall, we observed that addition of FP1 to both vaccine formulations was driving the increase in some lung T cell subsets (CD4 and CD8 TEMs and TRMs) suggesting that the observed increase may be antigen-specific, though it was notable that the Nano group had a higher frequency of CD103+ CD8 TRM (**Supplementary Figures 3A, B**).

**Figure 3:**
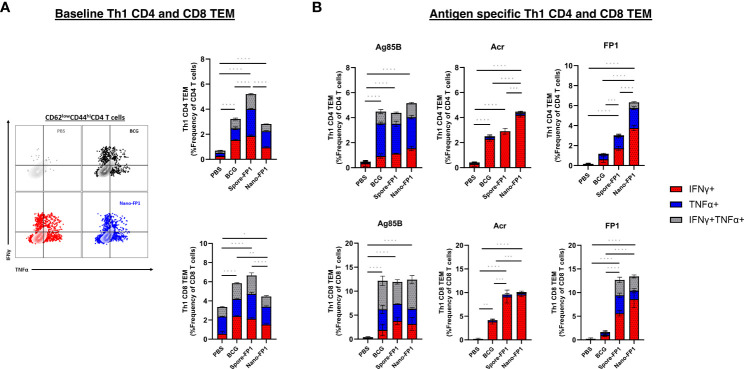
Th1 cytokine profile of CD4 and CD8 TEMs in the lungs after intranasal immunisation with Spore-FP1 and Nano-FP1. Lungs collected from mice three weeks after immunisation were processed to obtain single cell suspensions. **(A)** Representative dot plots and bar graph showing baseline CD4 and CD8 Th1 cytokine secretion by PBS, BCG, Spore-FP1 and Nano-FP1 groups. **(B)** Cells were plated and stimulated with either Ag85B, Acr or FP1. After 24 hours, cells were surface stained for T cell markers and intracellular IFNy and TNFα. Bar graphs showing antigen specific Th1 cytokine secretion by CD4 and CD8 T cells in the four groups. All bars are represent means +/- SEM. One-way ANOVA followed by Tukey’s multiple comparisons test was used to compare the groups. P<0.05 = *; P<0.01 = **; P<0.001 = ***; P<0.0001 = ****.

Th1 cytokines, IFNγ and TNFα, are known drivers of protective immune responses against Mtb (20, 21). As such, we also probed for Th1 cytokine-positive cells in the lungs of mice using intracellular cytokine staining for IFNγ and TNFα (**Supplementary Figure 1A**). **Figure 3A** shows that mice that received Spore-FP1 had significantly greater baseline Th1 cytokine-positive CD4 TEM cells than both BCG and Nano-FP1 groups (P<0.0001), and CD8 TEM cells against the Nano-FP1 (P<0.0001) but not the BCG group. No significant difference was seen in bystander CD4 or CD8 TEM between Nano-FP1 and BCG groups.

Since we were interested to investigate antigen specific Th1 responses, single cell suspensions of cells obtained from the lungs were spiked with whole antigens (Ag85B or Acr) or fusion protein. We saw that for both CD4 and CD8 TEM subsets, neither Spore-FP1 nor Nano-FP1 significantly increased Ag85B-specific Th1 cytokine positive cells (**Figure 3B**). However, we observed that Nano-FP1 had significantly greater Th1 Acr-specific CD4+ TEM than both Spore-FP1 (P<0.001) and BCG (P<0.0001) groups, and both Spore-FP1 and Nano-FP1 immunised animals had significantly greater frequencies of Th1 Acr-specific CD8+ TEM then the BCG group (P<0.001) (**Figure 3B**). In addition, FP1-specific Th1 CD4 and CD8 TEM cells were markedly higher in the Spore-FP1 and Nano-FP1 groups as compared to the BCG (P<0.001) (**Figure 3B**). It is also worth noting that Nano-FP1 immunised animals generated significantly higher frequencies of FP1-specific Th1 CD4+ TEM cells than the Spore-FP1 group (P<0.001) (**Figure 3B**). The increase in Ag85-, Acr- and FP-1 specific Th1 cytokine positive CD4 and CD8 TEMs were not seen in animals that were given Spore or Nano alone (**Supplementary Figures 4A, B**). Instead, we observed that the Spore and Nano groups had significantly lower Ag85B- and Acr-specific CD4TEMs as compared to either BCG or Spore/Nano-FP1 groups.

All these demonstrate that intranasal immunisation with Spore-FP1 and Nano-FP1 increases the frequency of potentially protective T cell populations even in the presence of strong mycobacterial immunity in the lungs.

### Systemic T cell immunity in mice after intranasal Spore-FP1 and Nano-FP1 immunisation

**Figure 4:**
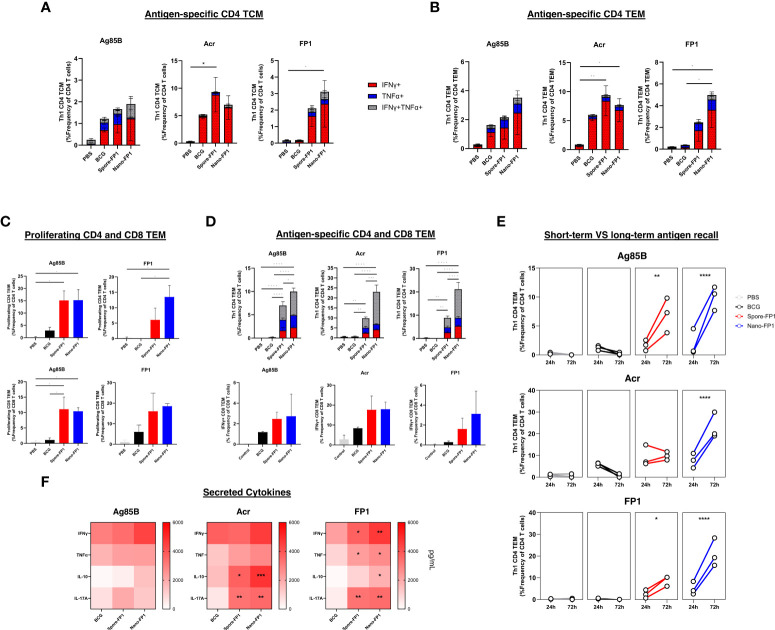
Systemic antigen-specific T cell responses after intranasal immunisation with Spore-FP1 and Nano-FP1. Spleens were collected from mice three weeks after immunisation and were processed to obtain single cell suspensions. Cells were plated and stimulated with either Ag85B, Acr or FP1. After 24 or 72 hours, samples surface stained for T cell markers and intracellular IFNγ and TNFα. Culture supernatants were also collected at 72hours to measure secreted Th1, Th2 and Th17 cytokines. Stacked bar graphs showing baseline antigen specific CD4 **(A)** TCM and **(B)** TEM with a Th1 profile after 24 hours of antigen stimulation. Bar graphs and stacked bar graphs showing **(C)** antigen-specific proliferating and **(D)** Th1 cytokine positive CD4 and CD8 TEM cells after 72 hours of stimulation. **(E)** Line graph depicting the frequency of antigen-specific Th1 CD4 TEM after 1 or 3 days of antigen stimulation in vitro **(F)** Heatmap showing IFNγ, TNFα, IL-10 and IL-17A cytokine secretion by splenocytes after antigen-stimulation. All bars represent means +/- SEM. One-way or two-way ANOVA followed by Tukey’s multiple comparisons test was used to compare the groups. P<0.05 = *; P<0.01 = **; P<0.001 = ***; P<0.0001 = ****.

We wanted to investigate the systemic immune responses in mice that were exposed to mucosal BCG infection, followed by a single intranasal vaccination with Spore-FP1 and Nano-FP1 by probing for antigen-specific central memory (TCM) and effector memory (TEM) CD4 T cells in the spleen. A short-term antigen recall assay was performed wherein splenocytes were incubated with the antigens for 24 hours to probe for baseline frequency of antigen-specific T cells in the spleen. **Figures 4A, B** both show that Spore-FP1 immunisation led to a significantly greater frequency of Acr-specific CD4 TCM and TEM. Meanwhile, Nano-FP1 immunised animals had significantly increased Acr-specific and FP1-specific CD4 TCM and TEM. Both vaccine candidates did not increase the frequency of Ag85B-specific CD4 TCM and TEM.

To investigate the capacity of T cells in the spleens to proliferate and survive sustained antigen stimulation, we also performed a long-term recall assay wherein splenocytes were stimulated with antigens for 72 hours. First, we observed greater frequencies of proliferating (Ki67+) CD4 and CD8 TEM cells among immunised groups after stimulation with Ag85B and FP1 (**Figure 4C**). More importantly, the frequency of Ag85B, Acr and FP-specific Th1 cytokine-positive CD4 and CD8 T cells were markedly higher among mice that received both vaccines (**Figure 4D**). This was coupled by an increase in the frequency of double positive (IFNγ+TNFα+) CD4 TEMs as compared to the predominantly IFNγ+ recall responses seen in the 24-hour antigen recall. Furthermore, the increase in magnitude of antigen specific Th1 CD4 TEMs was significantly higher among immunised groups after 3 days of antigen stimulation, most notably for Nano-FP1 immunised mice (**Figure 4E**).

Supernatants of splenocytes stimulated with Ag85B, Acr or FP1 for 72 hours were obtained to measure the levels of secreted IFNγ, TNFα, IL-10 and IL-17A (**Figure 4F**). We observed that although ICS staining revealed high frequencies of Th1 cytokine positive cells, the levels of secreted IFNγ and TNFα were not significantly different between BCG groups and immunised groups. In contrast, levels of secreted IL-10 and IL-17A were significantly higher among mice that received Spore-FP1 and Nano-FP1.

Together, these data suggest that intranasal immunisation with Spore-FP1 and Nano-FP1 generated Th1, Th2 and Th17 cytokine responses, all important drivers of anti-mycobacterial protection.

### Antigen-specific immunoglobulins in serum and BALF are increased after immunisation with Spore-FP1 and Nano-FP1

**Figure 5:**
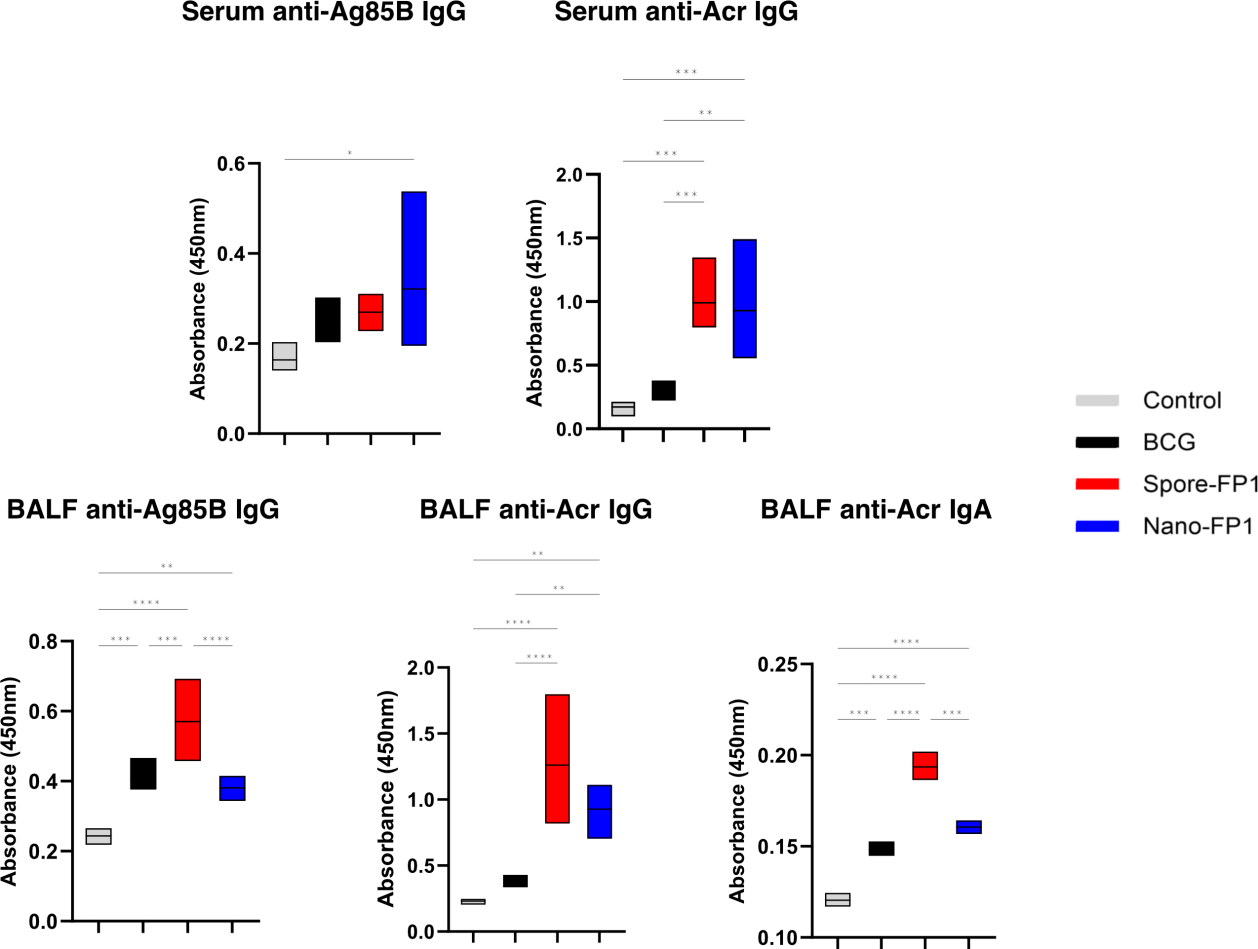
Systemic and mucosal antigen-specific immunoglobulins increase after immunisation with vaccine candidates. Serum and BAL fluid were obtained from mice to measure antigen-specific IgA and IgG response in the systemic and mucosal compartments, respectively. Box plots showing the levels of Ag85B- and Acr- specific IgG and IgA in the serum and BALF of mice after immunisation with Spore-FP1 and Nano-FP1. Box plots cover minimum to maximum values obtained with the assay. Dilutions of 1:20 is shown for serum anti-Ag85B and -Acr IgG, 1:2 for BALF anti-Ag85B and -Acr IgG, and 1:3 for BALF anti-Acr IgA. One-way ANOVA followed by Tukey’s multiple comparisons test was used to compare the groups. P<0.05 = *; P<0.01 = **; P<0.001 = ***; P<0.0001 = ****.

Antibodies have been suggested to provide some degree of protection against Mtb infection (22). We measured Ag85B- and Acr- -specific IgG and IgA in the serum and BALF of immunised mice (**Figure 5**). Looking at the systemic compartment, we observed that levels of Ag85B-specific IgG levels were significantly increased in the Nano-FP1 group (P<0.05) as compared to the control group but not the BCG group. Acr-specific IgG was significantly increased for both Spore-FP1 and Nano-FP1 immunised animals as compared to the BCG group. No detectable levels of circulating Ag85B- or Acr-specific IgA was seen (Data not shown). At the mucosal compartment, Spore-FP1 immunised animals had levels of Ag85B-specific IgG, Acr-specific IgG and Acr-specific IgA that were significantly greater than both BCG and Nano-FP1 groups (P<0.001). On the other hand, levels of these antibodies were not significantly higher than the BCG group among Nano-FP1 immunised mice apart from Acr-specific IgG (P<0.01). We did not detect levels of anti-Ag85B IgA in BALF (Data not shown). These data indicate that both vaccine candidates are able to boost antigen-specific immunoglobulins in the both the mucosal surface of the respiratory tract and in the systemic circulation.

### Poly(I:C) improves the adjuvanticity of inactivated spores and YC-NaMA in human monocyte-derived dendritic cells

**Figure 6:**
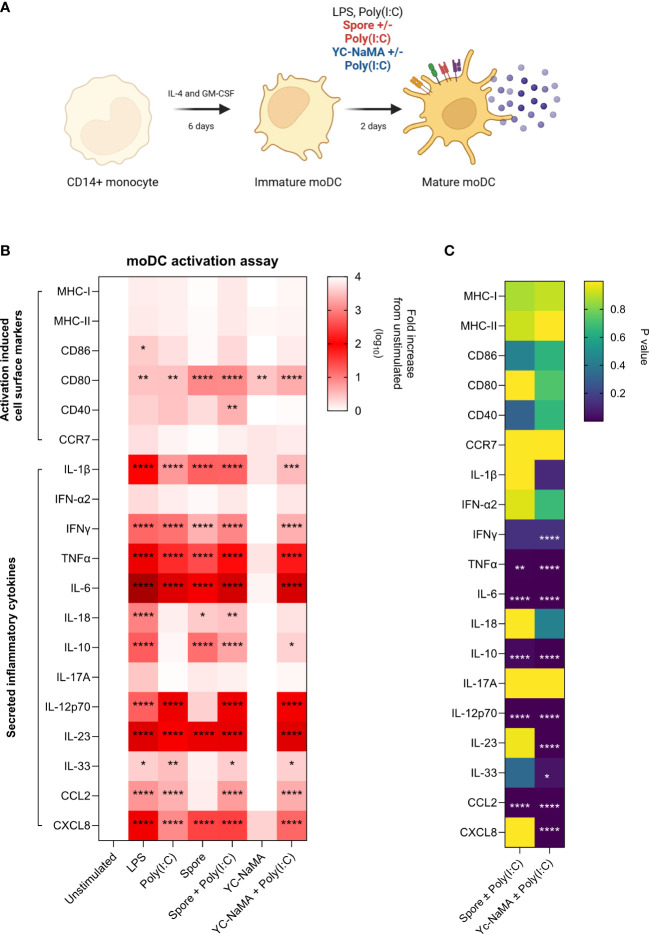
Poly(I:C) improves the adjuvanticity of inactivated spores and YC-NaMA in human monocyte-derived dendritic cells. **(A)** CD14+ monocytes were magnetically enriched from healthy donor PBMCs After 6 days of differentiation, moDCs were either left unstimulated, or treated with LPS, poly(I:C) or vaccine delivery systems with or without poly (I:C). After 48 hours, cells were surface stained for activation induced markers (MHC-I, MHC-II, CD80, CD86, CD40 and CCR7) for FACS analysis. Culture supernatants were also obtained to quantify secreted inflammatory cytokines (IL-1β, IFN-α2, IFNγ, TNFα, IL-6, IL-18, IL-10, IL-17A, IL-12p70, IL-23, IL-33, CCL2 and CXCL8). **(B)** Heatmap showing the log10 fold increase in cell-surface and secreted soluble mediators for each treatment group relative to unstimulated group. **(C)** Heatmap showing P values comparing YC-Nama or Spore with and without Poly(I:C). Data is obtained from the average of two healthy donors. Two-way ANOVA followed by Tukey’s multiple comparisons test was used to compare the groups. Asterisks depicted in A are significant differences from unstimulated. Asterisks in B show significant difference between two groups. P<0.05 = *; P<0.01 = **; P<0.0001 = ****.

As the vaccine formulations contain the delivery systems (Spore or YC-NaMA) and poly(I:C) as adjuvant, we designed an experiment to test these combinations in human monocyte-derived dendritic cells to specifically address the role of vaccine components interacting with innate immune cells. CD14+ cells from healthy donor PBMCs were differentiated into immature moDCs for 6 days. After incubation with delivery systems with or without poly(I:C), we measured activation-induced cell surface markers (MHC-I, MHC-II, CD80, CD86, CD40 and CCR7) as well as secreted soluble inflammatory mediators (IL-1β, IFN-α2, IFNγ, TNFα, IL-6, IL-18, IL-10, IL-17A, IL-12p70, IL-23, IL-33, CCL2 and CXCL8) (**Figure 6A**). Results showed that poly(I:C) improved the adjuvanticity of the delivery systems as shown by increased cell surface expression of activation-induced markers as well as secreted soluble mediators (**Figure 6B**). Similar to what we reported in mice, inactivated spores had strong intrinsic adjuvant properties as evidenced by upregulation in many cell surface markers and soluble mediators even in the absence of poly(I:C). In contrast, YC-NaMA strongly benefited from the addition of poly(I:C) as shown by significant increases in the secretion of several soluble mediators (IFNγ, TNFα, IL-6, IL-10, IL-12p70, IL-23, IL-33, CCL2 and CXCL8) (**Figure 6C**). We thus demonstrated that the addition of poly(I:C) to the vaccine formulation upregulates important markers and soluble mediators of dendritic cell activation, likely contributing significantly to the observed immunogenicity in mice.

### PBMCs from individuals with latent TB infection have greater immunoreactivity to FP1

**Figure 7:**
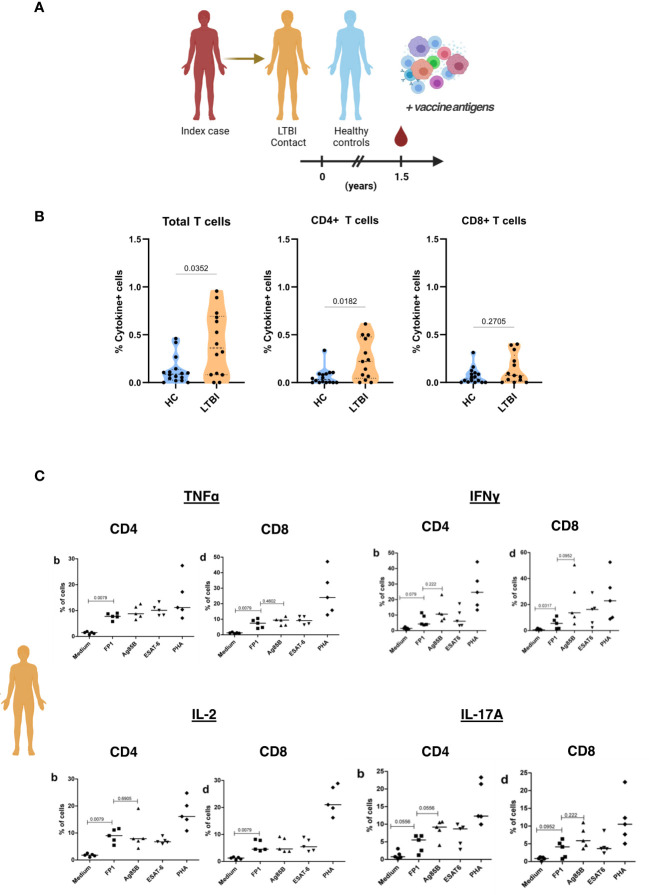
PBMCs from individuals with latent TB show immunoreactivity to FP1. **(A)** Household contacts of individuals with active TB (index case) were designated as either LTBI contacts or healthy controls based on IGRA positivity. PBMCs collected from these household contacts were treated with FP1 to ascertain CD4 and CD8 T cell immunoreactivity. **(B)** Truncated violin plots show Th1 cytokine (TNFα and IFNγ) positive T cells, CD4 T cells and CD8 T cells of HC and LTBI contacts. **(C)** Dot plots showing cytokine-secreting (TNFα, IFNγ, IL-2 and IL-17A) cells of LTBI contacts after stimulation with different antigens. Mann-Whitney test was used to compare HC and LTBI. One-way ANOVA followed by Tukey’s multiple comparisons test was used to compare different treatment groups for **(B)**.

Since we want to assess these vaccine candidates as potential post-exposure vaccines, we tested immunoreactivity of T cells obtained from individuals with LTBI to the principal vaccine antigen, the fusion protein FP1. Household contacts of individuals with active TB were recruited and designated as either LTBI contacts or healthy controls based on IGRA positivity. PBMCs from HC and LTBI contacts were then cultured with FP1 for 24 hours (**Figure 7A**). Results showed that LTBI contacts had significantly higher frequency of Th1 cytokine positive T cells than the healthy controls (**Figure 7B**). We observed that this difference can be attributed to CD4+ T cells (p=0.0182) but not CD8+ T cells (p=0.2705). We also looked into the presence of cytokine-secreting (TNFα, IFNγ, IL-2 and IL-17A) cells in a subset of LTBI contacts after stimulation with FP1 and Ag885B. We observed that LTBI contacts have higher frequency of TNFα, IFNγ, IL-2 and IL-17A secreting cells in the PBMCs than the non-exposed controls (**Figure 7C**). However, we did not observe any difference between Ag85B and FP1 stimulated PBMCs suggesting that FP1-specific responses were predominantly Ag85B specific.

## Discussion

Pulmonary TB is caused by the failure of localised lung immunity to restrict Mtb growth. During latent infection, a coordinated effort between both the innate and adaptive arms of the immune system prevents bacterial dissemination within the lungs. In some cases however, there is a breakdown in the capacity of immune effectors to “wall off” bacteria, eventually leading to active disease (23). One area of interest in the TB vaccine field is whether the mucosal delivery of vaccines to lungs that have existing mycobacterial immunity can increase populations of potentially protective T cell subsets systemically, and, importantly, locally. Several studies in mice have explored the use of post-exposure vaccines either as a stand-alone intervention (24) or as an adjunct to antibiotic therapy (25–30). Of these, one that tested mucosal vaccine delivery of an adenovirus vectored vaccine resulted in significantly increased antigen-specific lung CD8 T cells and markedly reduced bacterial burden after withdrawal of antibiotic therapy (30). We have previously shown that both Spore-FP1 and Nano-FP1 generate favourable immune responses and, more importantly, protection against aerosol Mtb challenge when used as a mucosal booster to parenterally administered BCG (17–19). In this study, we explored the immunological consequence of mucosal administration of both vaccines in mice with existing lung mycobacterial immunity. In our experiments, we used BCG to model lung mycobacterial immunity in LTBI individuals since they are a key population for post-exposure vaccination. Previously, it was shown that aerosol Mtb infection and mucosal BCG vaccination display similar trends in terms of kinetics of T cell immunity such as Ag85B+ tetramer-positive T cells in the lungs (16). Additionally, BCG has been shown to reside in the lungs for as long as 5 months after mucosal inoculation (31).

T cells are important mediators for the control of mycobacterial infection (32). Recent evidence has highlighted the importance of polyfunctional lung T cell subsets in controlling pulmonary Mtb infection in mice (33) and non-human primates (NHPs) (34, 35). Tissue resident memory T cells are key effector cells at sites of viral and bacterial infections owing to their capacity to respond faster through rapid proliferation and cytokine secretion upon receiving queues from the local environment (36). In mice, adoptive transfer of airway T cells obtained from the respiratory tract of mice with lung mycobacterial T cell immunity conferred significant reductions in lung bacterial load after pathogenic Mtb challenge (33, 37). Although there is still no broad consensus on how to phenotypically define tissue residency of T cells, it has been shown that cell surface markers of leukocyte activation and retention (CD69 and CD103), and leukocyte homing (CD62L) are useful for identifying TRM subsets (36, 38). In our study, we used flow cytometry to define lung T cell subsets based on the cell surface expression of these markers. We have observed that immunisation with Spore-FP1 and Nano-FP1 significantly increased CD4 TEM and CD8 TRM cells in the lungs. The upregulation in CD8 T cell subsets in the lungs is very encouraging since it’s been shown that activated CD8 T cells correlate with TB disease control in NHPs (39). It is interesting to note that for both CD69+ and CD69+CD103+ CD4 TRM subsets, we did not see a significant boosting effect by the candidate vaccine which suggests that boosting these specific subsets with one dose may not be sufficient, or the vaccine candidates we used are better at boosting CD8 T cell responses.

The production of Th1 cytokines, IFNγ and TNFα, are crucial for the control of Mtb infection in the lungs. Mice that lack or are depleted of these important cytokines prior to pathogenic Mtb challenge rapidly succumb to infection (20, 21). In humans, antibody-based blockade of TNFα increases the risk of TB reactivation by up to 25-fold (40). More importantly, recent studies in NHPs reveal that airway CD4 T cells with the capacity to produce both IFNγ and TNFα after intravenous BCG administration was predictive of protection against tuberculosis (35), while the presence of enriched populations of T cells with a Type 1 immune response correlate with bacterial control in the granuloma (41). Our results show that intranasal immunisation can be used as a strategy to increase the frequency of Th1 producing CD4 and CD8 T cells in the lungs. Interestingly, Ag85B-specific CD4 and CD8 T cells were not further increased by either candidate vaccines despite an increase in the frequencies of Acr- and FP-1 specific lung T cells.

In addition to lung mucosal immunity, T cell responses in secondary lymphoid organs also play a vital role in controlling mycobacterial infection since they are rich sources of pathogen-specific memory T cells capable of homing to the site of infection (42). Indeed, it has been shown that strategies that delivered vaccines parenterally led to the generation of protective memory T cells in the spleen (43, 44). Here, we showed that intranasal infection with BCG generates mycobacteria specific CD4 TEM and TCM, with frequencies of Acr-specific, but not Ag85B-specific, Th1 CD4 TCM and TEM populations increased by a single dose of both candidate vaccines. Notably, immunisation led to the generation of Ag85B and Acr-specific T cell responses that were able to proliferate after sustained antigen stimulation *in vitro*. This was similar to previous findings where immunisation strategies increased the frequency of non-terminally differentiated antigen-specific T cell subsets with better proliferative and cytokine producing capacity (45, 46). Apart from Th1 mediated immune responses, Th17 cells also play important roles in immunity against Mtb. Th17 cells are important immune mediators through their involvement in neutrophil recruitment, granuloma formation and maturation, and Th1 cell recruitment to the site of mycobacterial infection (47, 48). Although we observed only marginal increases in the levels of secreted IFNγ and TNFα, we saw elevated levels of IL-17A, a key Th17 cytokine, in spleens of immunised mice stimulated with Ag85B, Acr and FP1. Additionally, we observed grater secretion of IL-10 among immunised mice. IL-10 is a prototypical regulatory cytokine which is canonically regarded to negatively affect protective immunity. However, previous evidence suggests that IL-10 synergises with Th1/Th17 responses for better T cell-mediated immune responses against TB, even correlating with sterilizing immunity within granulomas (34, 49).

Immunisation with both Spore-FP1 and Nano-FP1 led to higher titres of antigen-specific antibodies in BALF and serum. Antibodies have controversial roles in TB but there is growing body of evidence that points to their value in anti-mycobacterial immunity through opsonisation, T cell activation, phagosome maturation or preventing bacterial dissemination (22). We, alongside others, have shown that passive transfer of antibodies is protective in mouse models of Mtb infection (50–53). Also, studies in NHPs demonstrated a correlation between BALF IgA with increased protection (34).

Proteins are not inherently immunogenic. As such, protein subunit vaccines are formulated with nanoparticles and immunostimulants as adjuvants. For intranasal vaccines, the addition of a nanoparticle-based delivery system can also dramatically improve the availability of the antigens to local antigen presenting cells (54). However, some nanoparticles are unable to drive dendritic cell maturation without the addition of immunostimulatns (55). In our formulations, we used inactivated *Bacillus subtilis* spores and Yc-NaMA as nanocarriers/delivery systems. Although we have shown that both nanocarriers increase the expression of key activation markers in mouse antigen presenting cells (18, 19), this was not verified in human APCs. In our animal studies, poly(I:C) was included to vaccine formulations to complement both delivery systems. Poly(I:C) is a double-stranded RNA (dsRNA)-like molecule that mimics viral dsRNA. After binding to Toll-like receptor 3 (TLR3) and melanoma differentiation-associated protein 5 (MDA5), it leads to increased IRF3 and NFκB signalling resulting in a broad range of proinflammatory effects (55). We observed that the addition of Poly(I:C) as immunostimulant led to a dramatic improvement in the cell surface activation and secreted mediator profile of moDCs, most especially for Yc-NaMA. It was notable that the secretion of IL-12p70 and IL-23, both important cytokines for the generation of Th1 and Th17 responses, respectively, were increased by Poly(I:C). Though not yet tested mucosally, in humans, poly-ICLC, a stabilised form of poly(I:C), was safe and well tolerated when given intramuscularly or subcutaneously (56).

Our antigen recall assays show that PBMCs from LTBI individuals have higher FP1-specific cytokine+ CD4 T cell responses than healthy controls. Individuals with previous exposure to mycobacteria, pathogenic or otherwise, exhibit systemic antigen specific T cell immunity, and probing for the presence of recall responses to vaccine antigens is a viable strategy for the preclinical assessment of candidate vaccines. FP1 is composed of Ag85B (Rv1886c), Acr (Rv2031c) and the heparin binding domain of HBHA (Rv0475) with the former two acting as protective antigen components of the vaccine, while HBHA is included to potentiate interactions with lung epithelium (57). Several studies have shown Ag85B and Acr responses in human TB cohorts. Ag85B specific CD4 T cell responses can be detected in PBMCs obtained from Mtb infected or BCG immunised individuals (58, 59). Importantly, it has been suggested that Ag85B specific T cells compartmentalise in the lungs due to higher observed frequencies of Ag85B-specific IFNγ responses in BALF compared to PBMC of latently infected household contacts (11). Meanwhile, contradictory evidence exists for Acr specific responses in humans cohorts where one study showed higher responses among LTBI individuals (60), but others did not see differences in responses between individuals with active TB disease, latent infection and healthy controls (61, 62).

A limitation of our study is the use of BCG instead of pathogenic Mtb. Inducing a LTBI-like state in mice has been done in the past through the Cornell Method (63) or as recently shown, the use of transgenic Mtb strains (13). However, given that latency is a relatively quiescent stage of mycobacterial infection in the lungs, we argue that both methods mentioned would also not be fully representative of LTBI due to the initial load of mycobacteria in the lungs of mice before antibiotic treatment. Additionally, the immunopathology caused by pathogenic Mtb in mice prior to antibiotic therapy may alter local lung homeostasis and function, further drifting the model away from a state of latent infection, where local processes closely resemble a healthier state. As such, we propose that the use of avirulent strains of mycobacteria such as BCG may serve as a viable strategy to model immune responses in a lung with latent TB infection.

In summary, post-exposure immunisation with Spore-FP1 and Nano-FP1 increased the frequency of desirable T cell and antibody responses in both mucosal and systemic compartments of mice. These favourable immune profiles suggest that Spore-FP1 and Nano-FP1 warrants further evaluation in the post-exposure model of Mtb infection, such as the Cornell method, to establish merit for further development as potential mucosal vaccines in humans with LTBI.

## Data availability statement

The raw data supporting the conclusions of this article will be made available by the authors, without undue reservation.

## Ethics statement

The studies involving humans were approved by Ministry of Health Committee of Bioethics and Health ref 298/CNBS/15 and St. George’s Hospital London under ethical approval SGREC16.0009. The studies were conducted in accordance with the local legislation and institutional requirements. Written informed consent for participation in this study was provided by the participants’ legal guardians/next of kin. The animal study was approved by from London School of Hygiene and Tropical Medicine Ethics committee. The study was conducted in accordance with the local legislation and institutional requirements.

## Author contributions

EV: Conceptualization, Formal analysis, Investigation, Methodology, Visualization, Writing – original draft, Writing – review & editing. AT: Data curation, Formal analysis, Methodology, Writing – review & editing. MK: Methodology, Writing – review & editing. TM: Resources, Writing – review & editing. MP: Methodology, Writing – review & editing, Supervision. TH: Supervision, Writing – review & editing. RR: Conceptualization, Funding acquisition, Investigation, Project administration, Resources, Supervision, Writing – review & editing.

## Supplementary material

Supplementary Figure 1 Gating strategy for probing T cell subsets (CD4 and CD8 TEM, TRM and Th1 cytokine positive cells) in the lungs of immunised mice.

Supplementary Figure 2
**(A)** Bar graphs of absolute cell counts and **(B)** stacked bars showing frequency of conventional T cells (CD4+ and CD8+) in the lungs of mice across different groups. One-way ANOVA followed by Tukey’s multiple comparisons test was used to compare different treatment groups. P<0.01 = **; P<0.001 = ***; P<0.0001 = ****.

Supplementary Figure 3 Frequency of CD4 and CD8 TEM/TRM cells in the lungs of mice after receiving **(A)** Spore/Spore-FP1 or **(B)** Nano/Nano-FP1. One-way ANOVA followed by Tukey’s multiple comparisons test was used to compare different treatment groups. P<0.05 = *; P<0.01 = **; P<0.001 = ***.

Supplementary Figure 4 Stacked bars showing the frequency of baseline and antigen-specific (Ag85B, Acr and FP1) IFNγ+, TNFα+ and IFNγ+TNFα+ cytokine positive CD4 and CD8 TEM cells in the lungs of mice after receiving **(A)** Spore/Spore-FP1 or **(B)** Nano/Nano-FP1. Two-way ANOVA followed by Tukey’s multiple comparisons test was used to compare different treatment groups. P<0.05 = *; P<0.01 = **; P<0.001 = ***.

Supplementary Figure 5 Gating strategy for probing T cell subsets in the spleen of immunised mice.

Supplementary Figure 6 Antigen-specific (Ag85B, Acr and FP1) Th1 intracellular cytokine expression of CD4 and CD8 TCM and TEM cells in the spleen of **(A)** Spore/SporeFP1 and **(B)**Nano/Nano-FP1 immunised mice after 24 hours of antigen recall. Two-way ANOVA followed by Tukey’s multiple comparisons test was used to compare different treatment groups. P<0.05 = *; P<0.01 = **; P<0.001 = ***.

Supplementary Figure 7 Antigen-specific proliferation and Th1 intracellular cytokine expression of CD4 and CD8 TEM cells in the spleen of **(A)** Spore/SporeFP1 and **(B)** Nano/Nano-FP1 immunised mice after 72 hours of antigen recall with Ag85B, Acr and FP1. Two-way ANOVA followed by Tukey’s multiple comparisons test was used to compare different treatment groups. P<0.05 = *; P<0.01 = **; P<0.001 = ***.
