# Supplementary figures and images for: Mucosal and systemic immune responses after a single intranasal dose of nanoparticle and spore-based subunit vaccines in mice with pre-existing lung mycobacterial immunity
Source: Front Immunol. 2023 Dec 7;14:1306449. doi: 10.3389/fimmu.2023.1306449 (PMC10733481; doi:10.3389/fimmu.2023.1306449)

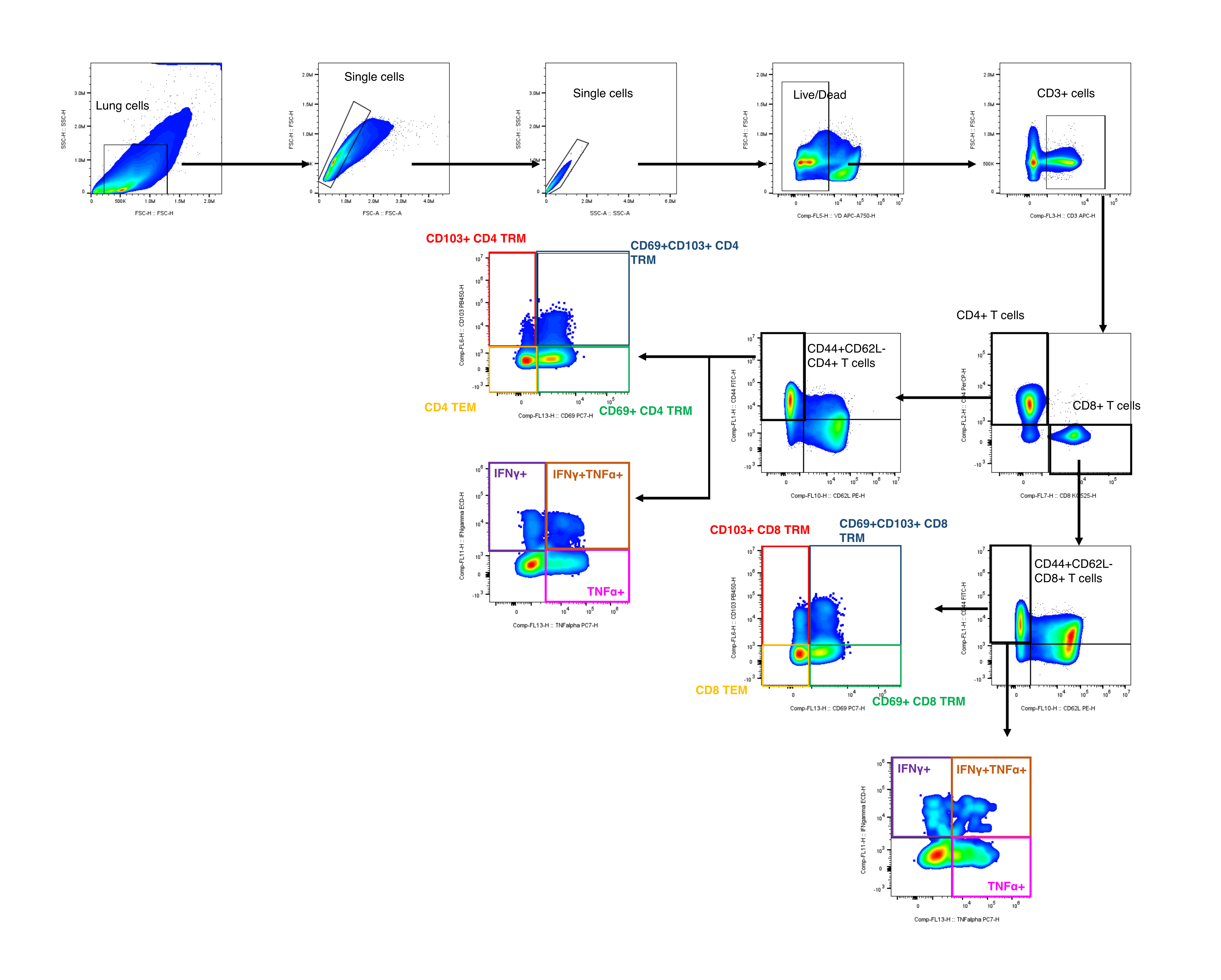

Supplement: Supplementary Figure 1 — Gating strategy for probing T cell subsets (CD4 and CD8 TEM, TRM and Th1 cytokine positive cells) in the lungs of immunised mice. [file Image_1.tiff]

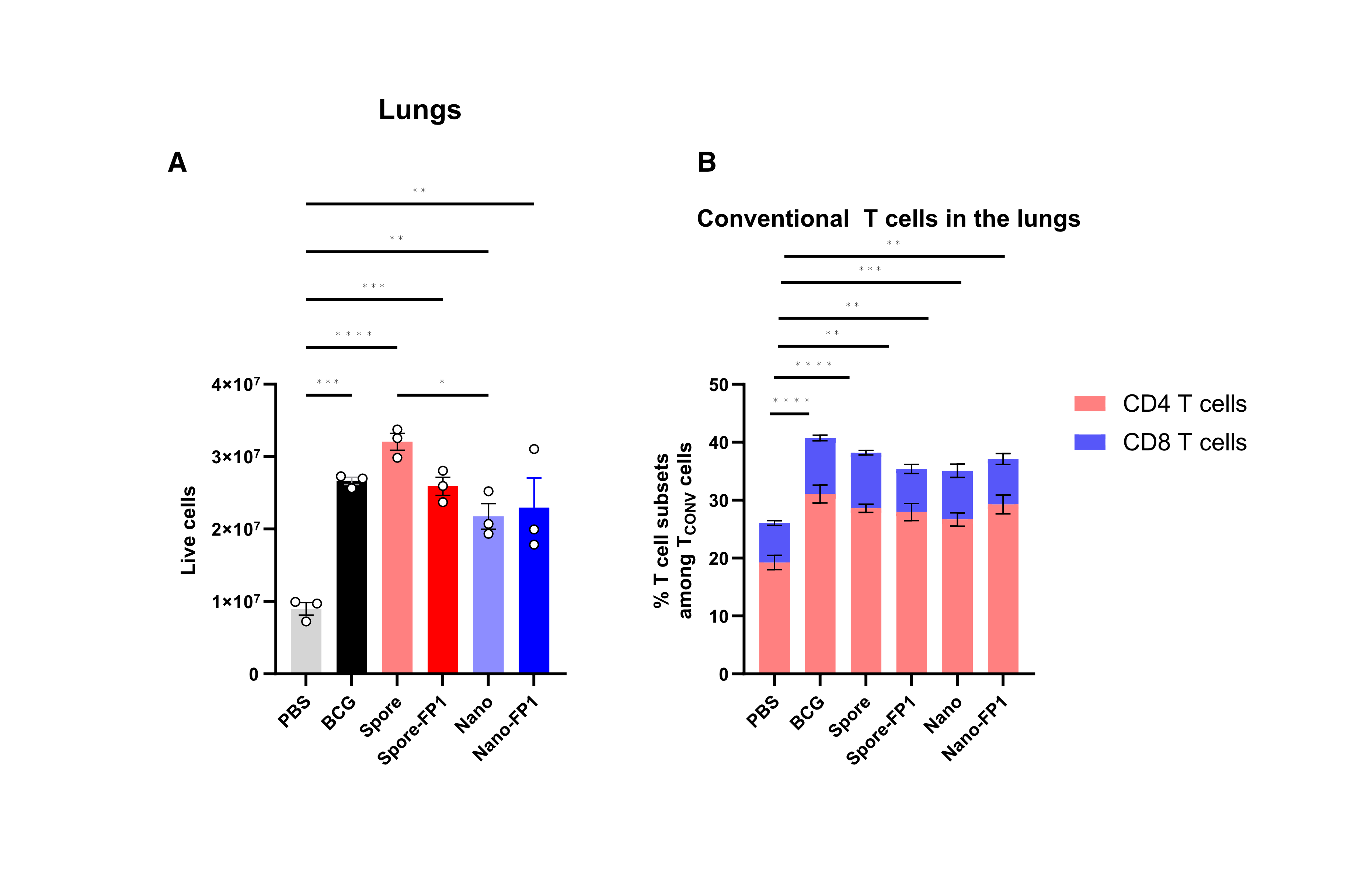

Supplement: Supplementary Figure 2 — (A) Bar graphs of absolute cell counts and (B) stacked bars showing frequency of conventional T cells (CD4+ and CD8+) in the lungs of mice across different groups. One-way ANOVA followed by Tukey’s multiple comparisons test was used to compare different treatment groups. P<0.01 = **; P<0.001 = ***; P<0.0001 = ****. [file Image_2.tiff]

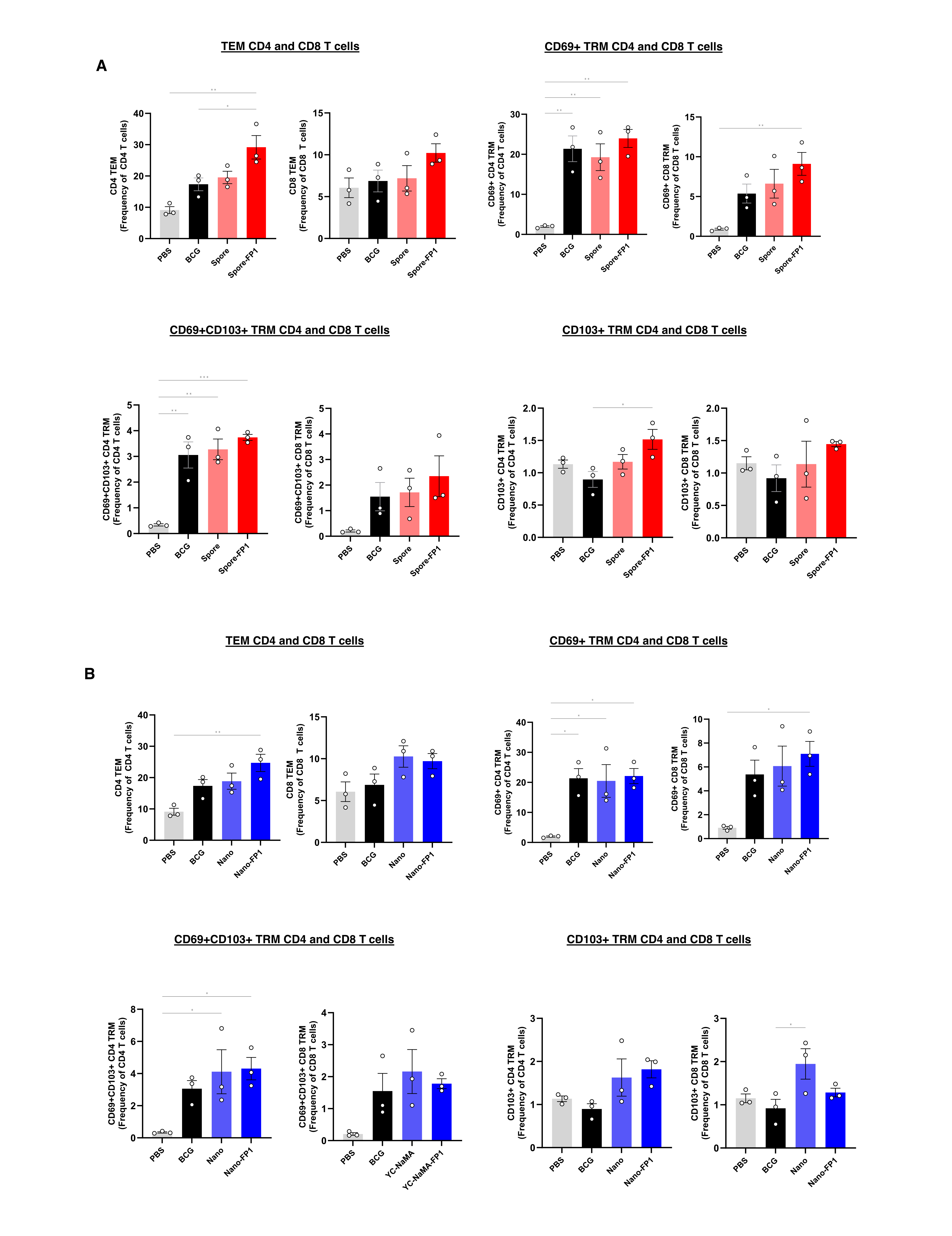

Supplement: Supplementary Figure 3 — Frequency of CD4 and CD8 TEM/TRM cells in the lungs of mice after receiving (A) Spore/Spore-FP1 or (B) Nano/Nano-FP1. One-way ANOVA followed by Tukey’s multiple comparisons test was used to compare different treatment groups. P<0.05 = *; P<0.01 = **; P<0.001 = ***. [file Image_3.tiff]

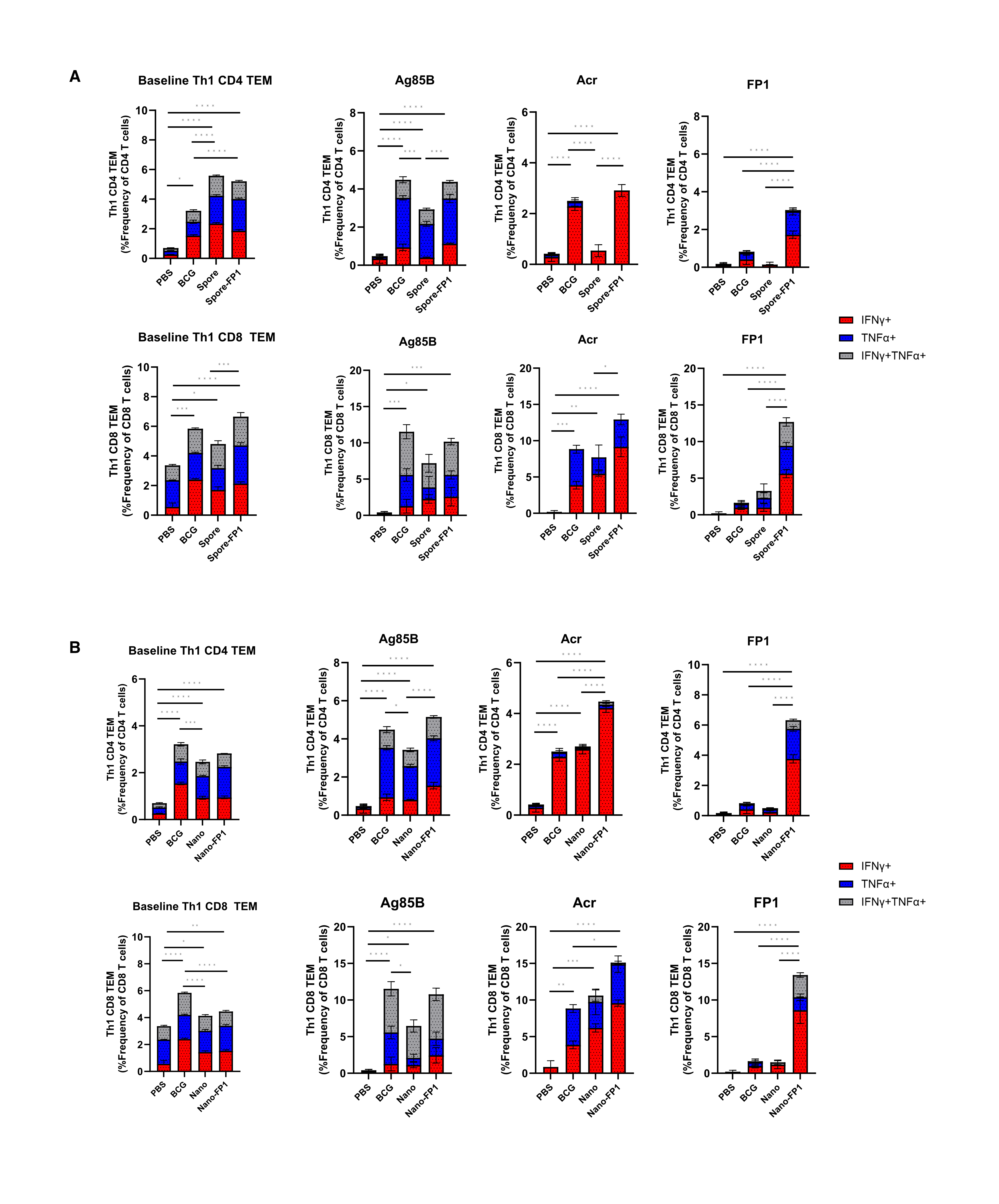

Supplement: Supplementary Figure 4 — Stacked bars showing the frequency of baseline and antigen-specific (Ag85B, Acr and FP1) IFNγ+, TNFα+ and IFNγ+TNFα+ cytokine positive CD4 and CD8 TEM cells in the lungs of mice after receiving (A) Spore/Spore-FP1 or (B) Nano/Nano-FP1. Two-way ANOVA followed by Tukey’s multiple comparisons test was used to compare different treatment groups. P<0.05 = *; P<0.01 = **; P<0.001 = ***. [file Image_4.tiff]

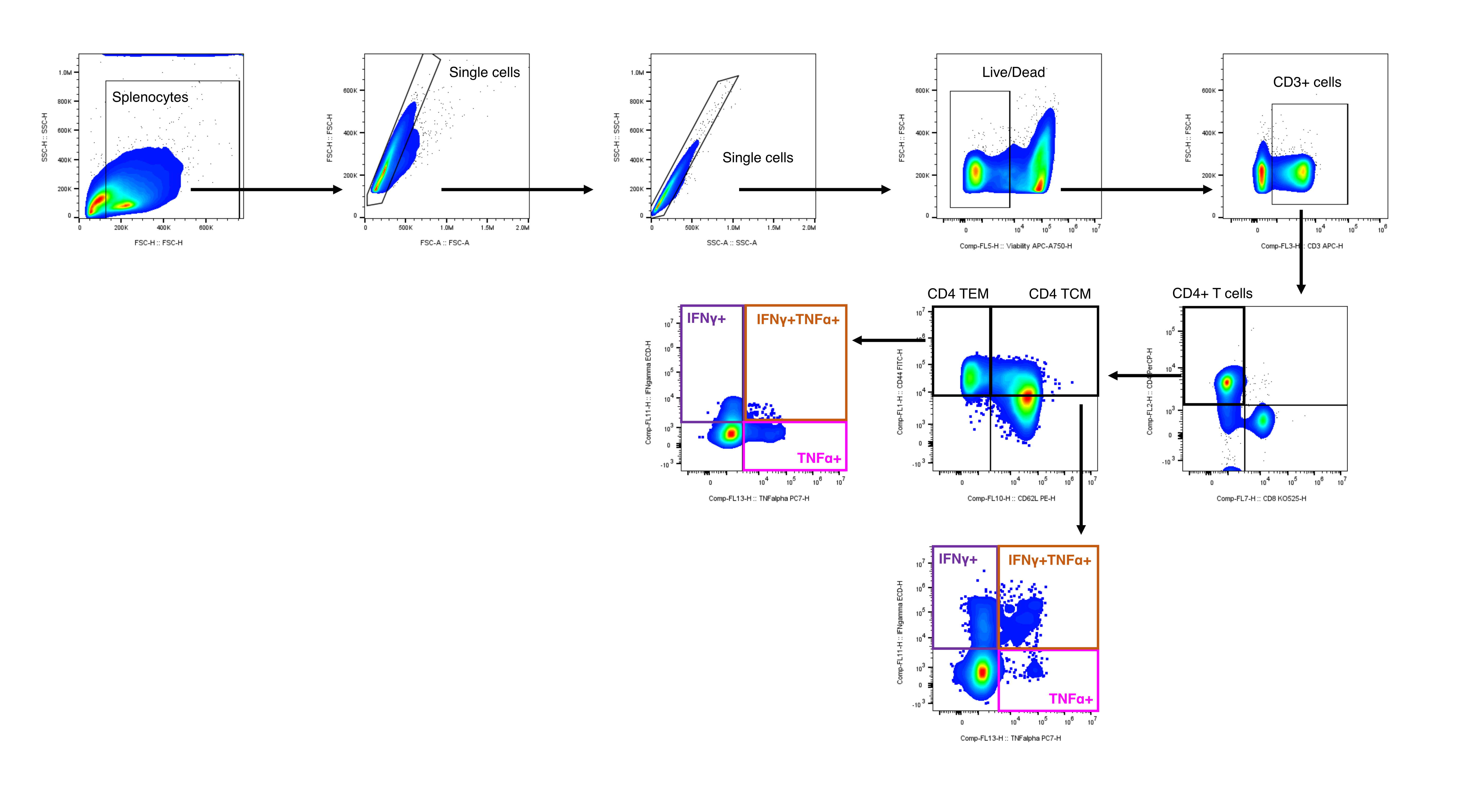

Supplement: Supplementary Figure 5 — Gating strategy for probing T cell subsets in the spleen of immunised mice. [file Image_5.tiff]

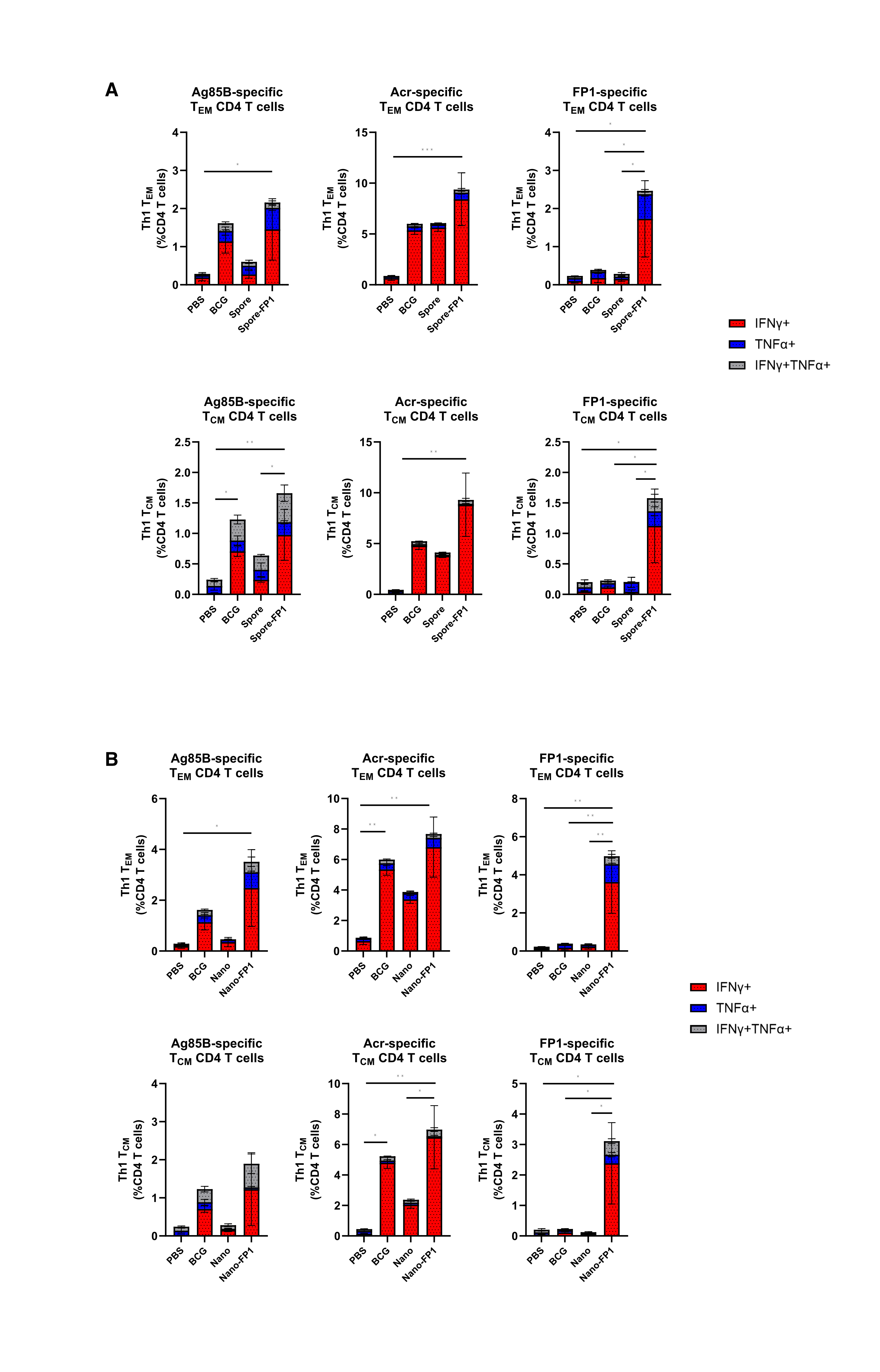

Supplement: Supplementary Figure 6 — Antigen-specific (Ag85B, Acr and FP1) Th1 intracellular cytokine expression of CD4 and CD8 TCM and TEM cells in the spleen of (A) Spore/SporeFP1 and (B)Nano/Nano-FP1 immunised mice after 24 hours of antigen recall. Two-way ANOVA followed by Tukey’s multiple comparisons test was used to compare different treatment groups. P<0.05 = *; P<0.01 = **; P<0.001 = ***. [file Image_6.tiff]

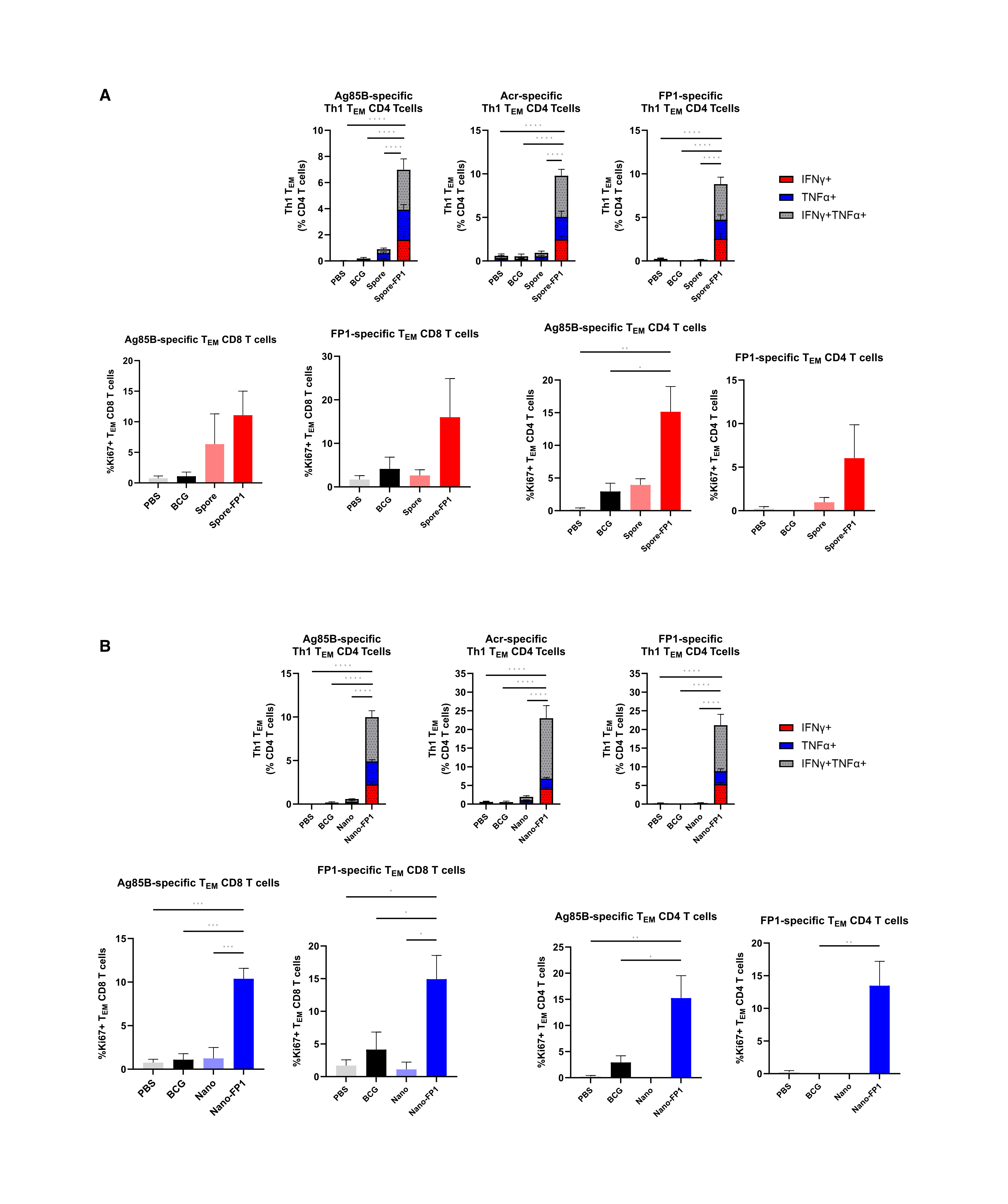

Supplement: Supplementary Figure 7 — Antigen-specific proliferation and Th1 intracellular cytokine expression of CD4 and CD8 TEM cells in the spleen of (A) Spore/SporeFP1 and (B) Nano/Nano-FP1 immunised mice after 72 hours of antigen recall with Ag85B, Acr and FP1. Two-way ANOVA followed by Tukey’s multiple comparisons test was used to compare different treatment groups. P<0.05 = *; P<0.01 = **; P<0.001 = ***. [file Image_7.tiff]
